# Treatment of Cardiovascular Disease in Rheumatoid Arthritis: A Complex Challenge with Increased Atherosclerotic Risk

**DOI:** 10.3390/ph15010011

**Published:** 2021-12-22

**Authors:** Saba Ahmed, Benna Jacob, Steven E. Carsons, Joshua De Leon, Allison B. Reiss

**Affiliations:** Department of Medicine and Biomedical Research Institute, NYU Long Island School of Medicine and NYU Langone Hospital, Long Island, Mineola, NY 11501, USA; Saba.Ahmed@NYULangone.org (S.A.); bjacob04@nyit.edu (B.J.); Steven.Carsons@NYULangone.org (S.E.C.); Joshua.DeLeon@NYULangone.org (J.D.L.)

**Keywords:** methotrexate, hydoxychloroquine, TNF-α, cholesterol, atherosclerosis, rheumatoid arthritis, cytokines, ABC transporters

## Abstract

Rheumatoid arthritis (RA) carries significant risk for atherosclerotic cardiovascular disease (ASCVD). Traditional ASCVD risk factors fail to account for this accelerated atherosclerosis. Shared inflammatory pathways are fundamental in the pathogenesis of both diseases. Considering the impact of RA in increasing cardiovascular morbidity and mortality, the characterization of therapies encompassing both RA and ASCVD management merit high priority. Despite little progress, several drugs discussed here promote remission and or lower rheumatoid disease activity while simultaneously conferring some level of atheroprotection. Methotrexate, a widely used disease-modifying drug used in RA, is associated with significant reduction in cardiovascular adverse events. MTX promotes cholesterol efflux from macrophages, upregulates free radical scavenging and improves endothelial function. Likewise, the sulfonamide drug sulfasalazine positively impacts the lipid profile by increasing HDL-C, and its use in RA has been correlated with reduced risk of myocardial infraction. In the biologic class, inhibitors of TNF-α and IL-6 contribute to improvements in endothelial function and promote anti-atherogenic properties of HDL-C, respectively. The immunosuppressant hydroxychloroquine positively affects insulin sensitization and the lipid profile. While no individual therapy has elicited optimal atheroprotection, further investigation of combination therapies are ongoing.

## 1. Introduction

Rheumatoid arthritis (RA) is a chronic immune-mediated inflammatory disorder that leads to the breakdown of immune tolerance [1]. It affects women disproportionately and usually presents between the ages of 40–60 [2,3]. Worldwide prevalence of RA is approximately 0.5–1% [4]. This disorder primarily manifests as joint pain, swelling and stiffness. Over time, uncontrolled inflammation leads to disease progression, irreversible destruction of cartilage and bone, and ultimately to reduced range of motion and severe physical disability [5]. Since RA is a systemic disease, extra-articular manifestations can affect multiple organ systems. Lung involvement is the most common, but vasculitis and rheumatoid nodules on the skin are also observed [6,7,8].

Dyslipidemia and atherosclerosis are major comorbidities associated with RA [9]. It is well established that RA confers increased risk for atherosclerotic cardiovascular disease (ASCVD), a chronic inflammatory and lipid-depository disease [10,11]. Traditional ASCVD risk factors fail to fully account for this increase, making it difficult to identify and treat these patients at the preclinical stage [12,13,14]. In comparison to the general population, RA patients have a CVD risk approximately 50% higher and a 1.6 times higher rate of acute myocardial infarction and ischemic stroke [15,16]. The risk of myocardial infarction is on a par with that of persons with diabetes mellitus [17].

ASCVD adds to the substantial burden on RA patients, contributing to overall morbidity, decline in physical function, and diminished quality of life [18,19].

## 2. Methods

An extensive literature review was performed using PubMed and Google with regard to the drugs used in rheumatoid arthritis and their cardiovascular effects. The search terms included “rheumatoid arthritis“, “atherosclerosis”, “rheumatoid arthritis AND atherosclerosis AND mechanism”, “cardiovascular disease”, “treatment”, “medications”, “DMARDs”, “methotrexate AND cardiovascular” and “TNF-α AND cardiovascular.” Studies available in English from 1989 onward were included. This yielded approximately 600 manuscripts from which we narrowed the scope by looking at human studies (observational, randomized, prospective and retrospective) as well as meta-analysis papers and reviews of mechanism of action of individual drugs. We excluded cancer, diabetes and autoimmune rheumatic disorders other than rheumatoid arthritis. The final number of papers reviewed after applying these selection criteria was 212. 

## 3. Background

### 3.1. Causes of Increased ASCVD Risk in RA

**Figure 1:**
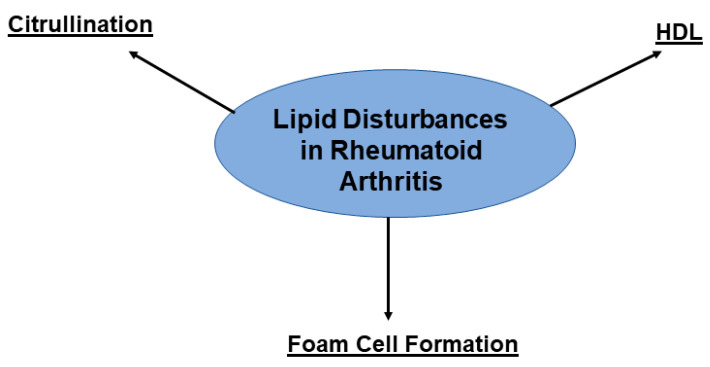
Pro-atherogenic disordered lipid transport and processing in RA. Both low density lipoprotein (LDL) and high density lipoprotein (HDL) are impacted by the inflammatory milieu in RA. Disruptions in lipid composition and transport observed in RA include (1) Citrullination. Citrullinated and homocitrullinated forms of LDL have atherogenic properties and may be abundant in RA plasma. (2) HDL abnormalities. Circulating levels of HDL may be low and paraoxonase 1 (PON1) activity of HDL impaired. Poorly functioning PON1 reduces antioxidant capacity of HDL. (3) Enhanced foam cell formation. Downregulation of cholesterol efflux proteins coupled with upregulation of scavenger receptors attenuates cholesterol outflow and increases oxidized LDL uptake within macrophages leading to lipid overload and formation of foam cells. TNF-α increases levels of oxidatively modified LDL via augmentation of reactive oxygen species generation.

Lipid disturbances are considered one of the primary factors underlying the increased risk for ASCVD in the RA population (Figure 1). Similar to the general population, circulating levels of high density lipoprotein (HDL) are inversely proportional to incidence of cardiovascular events, but beyond quantity, HDL functionality also affects ASCVD risk in RA [20,21,22,23]. Under normal circumstances, HDL is highly atheroprotective, acting as an antioxidant while facilitating cholesterol efflux [24]. However, these functions are impaired in the RA setting [25]. Paraoxonase (PON)-1 activity, the component responsible for HDL antioxidant properties, is decreased in RA correlating with an increased carotid plaque burden [26,27,28]. HDL in patients with high disease activity has also been found to have reduced cholesterol efflux capacity [29,30,31]. This reduced efflux capacity may result from impaired binding of ATP to the ATP binding cassette transporter (ABC)G1 [32]. 

In addition, RA plasma can foster a combination of attenuated cholesterol outflow and increased scavenger receptor-mediated oxidized low density lipoprotein (LDL) uptake by monocytes and macrophages via the downregulation of a set of cholesterol efflux proteins (ABCA1, ABCG1, 27-hydroxylase) coupled with the upregulation of scavenger receptors (CD36, LOX1 and CXCL16). This is a highly favorable setting for macrophage cholesterol overload and foam cell formation [33,34,35]. Changes in LDL composition resulting in greater levels of glycated end-products, oxidation, enhanced phagocytosis, and fatty acid accumulation have also been observed [36,37,38]. Citrullinated and homocitrullinated forms of LDL have atherogenic properties and may also be present in RA plasma [39]. Further, Lipoprotein a (Lp(a)), an atherogenic lipoprotein complex containing apolipoprotein (apo)A and apo B, is found at high levels in RA and is predictive of increased ASCVD risk [40]. A deeper understanding of these aberrations in lipoprotein function in rheumatoid arthritis patients may provide insight into methods for alleviating the cardiovascular burden.

Damage to endothelium occurs early in the atherosclerotic process and leads to endothelial dysfunction and activation, upregulation of adhesion molecules and interruption of the monolayer. The pro-inflammatory cytokine tumor necrosis factor (TNF)-α, produced in excess in RA, causes the release of adhesion molecules and is one factor that drives endothelial dysfunction [41]. Damaged endothelium allows oxidized LDL (oxLDL) and inflammatory cells to accumulate in the arterial intima where monocyte-to-macrophages differentiation, oxLDL internalization, and foam cell formation occur [42,43]. A cross-sectional study of 50 newly diagnosed early RA patients by Salem et al. [42] found that serum levels of vascular cell adhesion molecule (VCAM)-1 were elevated and correlated with disease activity and oxidative stress. VCAM-1 levels, considered an indicator of endothelial dysfunction, were significantly lower in treated patients compared to newly diagnosed patients. Higher serum levels of a soluble form of VCAM-1 are associated with accelerated progression of subclinical atherosclerosis [44].

In RA, damage to endothelium can also occur due impaired functioning of endothelial nitric oxide synthase which reduces availability of nitric oxide, a potent anti-inflammatory and vasodilator [45,46]. RA patients have high circulating concentrations of the methylated arginine asymmetric dimethylarginine (ADMA), a potent endogenous inhibitor of endothelial nitric oxide synthase [47].

### 3.2. RA and CVD: Shared Pathways

Development of CVD is a life-threatening systemic consequence of RA [48,49,50]. While chronic inflammation, a hallmark of RA, is likely responsible for much of the elevated CVD risk, traditional CVD risk factors such as obesity, diabetes, smoking and hypertension are over-represented in RA as well [51,52,53,54,55,56]. As the strong link between the two disorders worsens prognosis, it is essential to explore their connection in order to minimize health burden in RA. Doing so reveals shared inflammatory pathways fundamental to both disease processes with many pro-inflammatory cytokines implicated in RA also playing a central role in ASCVD [20,57,58,59]. Most prominent of the cytokines involved in both RA and ASCVD are TNF-α, interleukin (IL)-6 and IL-1β [60,61]. Elevated concentrations of each of these three cytokines have been correlated to the presence of coronary artery calcification, a severe complication of the atherosclerotic process [62,63,64,65]. A critical inflammatory pathway involved in the formation of atherosclerotic plaque is the oxLDL-stimulated production of a broad spectrum of cytokines by macrophages, with TNF-α and IL-1β playing major roles [66]. 

TNF-α is a key pro-inflammatory mediator in the pathogenesis of RA that promotes the development of atherosclerosis [67]. Elevated serum TNF-α is associated with dyslipidemia in RA patients. The cytokine not only impairs the anti-oxidant effects of HDL, but also promotes the formation of oxLDL within the arterial intima [68,69,70]. 

TNF-α has been linked to vascular injury in both acute and chronic inflammatory states. It promotes endothelial expression of chemokines and adhesion molecules that bring about leukocyte adherence and migration into the intimal layer of the arterial wall [71,72]. TNF-α also causes endothelial apoptosis leading to breakdown of endothelial barrier integrity and a pro-thrombotic environment [73,74]. Nitric oxide and thrombomodulin production are decreased in the setting of elevated TNF-α, further encouraging thrombotic events [71,75]. The inflammatory milieu of RA promotes formation of atherosclerotic plaques that are unstable and vulnerable to rupture [76,77]. 

IL-1β is a secretory protein produced by macrophages and endothelial cells. It induces endothelial cells to express surface adhesion molecules that foster recruitment of macrophages into the arterial intima [78]. 

Numerous additional inflammatory cytokines foster a pro-atherogenic, pro-thrombotic state, including IL-17, IL-18, and IL-33 [20,79,80,81]. Many of these cytokines act in synergy with each other and are involved in multiple signaling events that occur simultaneously [82]. Of the myriad mediators, nuclear factor (NF)-κB, stands out as a “master switch” that turns on multiple pro-atherosclerotic genes, including VCAM-1, intercellular adhesion molecule-1, and macrophage-colony-stimulating factor and induces vascular inflammation [83,84]. NF-κB stimulates production of C-reactive protein, IL-6, and TNF-α [85,86].

Serum amyloid A (SAA), an acute-phase protein found at elevated levels in RA [87]. SAA is linked to atherosclerosis by its ability to bind to HDL and attenuate its anti-oxidative properties [88,89].

**Figure 2:**
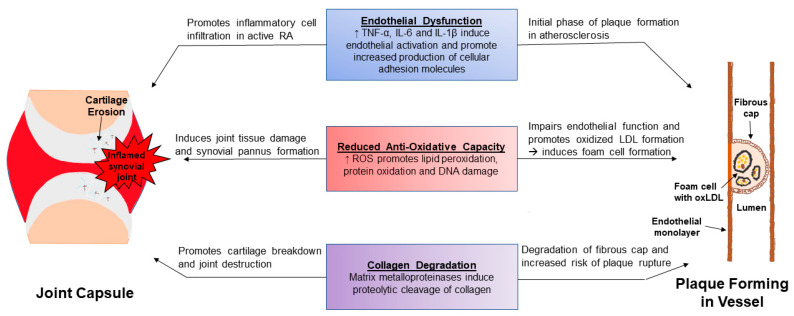
Shared inflammatory pathways are fundamental in the development of rheumatoid synovitis and ASCVD. The initial phase involves endothelial dysfunction which promotes inflammatory cell infiltration within the joint capsule and the inception of plaque formation within the sub-intima of arteries. The reduction in anti-oxidative capacity in RA patients not only accelerates joint tissue damage, but also promotes LDL oxidation and foam cell formation. Increased matrix metalloproteases (MMPs) evident in RA plasma then stimulates cartilage degradation in the joint and initiate deterioration of the fibrous cap surrounding the atherosclerotic plaque. This encourages plaque instability and eventual rupture which can lead to acute myocardial infarctions and strokes, depending on vessel location.

In RA, matrix metalloproteinases (MMPs), which degrade collagen as well as non-collagen components of the joint extracellular matrix, are produced by invading synovial pannus. Serum concentrations of MMP-1 and MMP-3 correlate with RA disease activity and predict progression of joint destruction [90,91,92]. MMPs are also associated with plaque instability and rupture [77,93,94] (Figure 2). Atherosclerotic plaque formation in RA may be accelerated due not only to MMPs, but also due to inflammatory cytokines which stimulate vascular smooth muscle proliferation and migration, leading to arterial intimal thickening, while also promoting plaque destabilizing microcalcification [95,96]. 

The CD40-CD40L axis, which mediates pro-inflammatory and pro-fibrotic pathways, is another shared process in both RA and ASCVD [97,98,99]. CD40, a member of the TNF receptor superfamily, is linked to cardiovascular events including myocardial infarction, plaque rupture and thrombosis [100,101,102]. 

Oxidative stress, the imbalance of reactive oxygen species generation and antioxidant defense, contributes to the pathogenesis of both RA and ASCVD [103,104,105]. Plasma antioxidant capacity is reduced in RA and oxidative stress increased [106,107]. Reactive oxygen species are chemically reactive free radicals that mediate tissue damage within joints and other body systems by inciting lipid peroxidation, protein oxidation and DNA damage [108,109]. Oxidative stress drives the atherosclerotic process and RA creates a source of excessive reactive oxygen species, thus contributing to a pro-atherosclerotic milieu [110]. Reactive oxygen species impair functionality of vascular endothelium and participate in oxidation of LDL, two critical processes involved in ASCVD [111]. In a cell culture model using ECV304, a spontaneously transformed cell line from human umbilical vein endothelial cells, plasma from RA patients was shown to reduce nitric oxide synthesis and increase reactive oxygen species production [112]. 

## 4. Treatment of RA—Does It Address CVD Risk?

Considering the significant impact on RA morbidity and mortality, the development of treatments that incorporate ASCVD management would be expected to merit high priority, but surprisingly little progress has been achieved. The current goal of RA therapy is remission or low disease activity, characterized as a state with minimal joint pain and swelling, prevention of joint deformities and radiographic damage, maintenance of quality of life and control of extra-articular manifestations [113,114,115] obtainable in approximately 10–50% of patients 

Given the substantial strain this chronic disease carries for both the individual and society, extensive research into its treatment has been conducted. DMARDs, which are slow-acting agents beneficial in improving the symptoms and radiographic progression, remain at the forefront of therapy [116,117,118]. Methotrexate has dominated the DMARDs as the medication of choice; however, alternatives include leflunomide, sulfasalazine and hydroxychloroquine. Numerous studies have led to the understanding that combination therapies are more effective than mono-therapies. Considering the significant role cytokines and other molecules play in the pathology of the disease, relatively newer treatments utilizing these biological targets have also emerged in recent times [116,117]. These drugs accomplish their goal via four main mechanism: blockade of TNF-α, inhibiting IL-6, blocking T-cell co-stimulation as well as depletion of B-cell. While non-steroidal anti-inflammatory drugs (NSAIDs) and glucocorticoids can relieve pain and inflammation, they do not prevent progressive joint damage and their long-term use may be harmful [118,119]. Ancillary therapies including physical activity, exercise, muscle strengthening and diets supporting a healthy microbiome are also implemented. In severe cases, when the damage is too advanced total joint replacement, most commonly of the hip or knee, may be required. 

The NIH funded clinical trial, Treatments Against RA and Effect on FDG-PET/CT (fluorodeoxyglucose-positron emission tomography with computed tomography) (TARGET, NCT02374021) [120], aims to determine whether anti-inflammatory DMARDs used to treat RA also reduced the risk of CVD. This will be accomplished by comparing two separate therapies, administration of methotrexate (MTX) combined with a TNF-α inhibitor or triple therapy with MTX, sulfasalazine and hydroxychloroquine. This paper explores the effects of cholesterol-lowering therapy and of the individual drugs used in TARGET on development of ASCVD as we move toward discovering which, if any, combinations confer optimal atheroprotection. 

## 5. ASCVD Management in RA

### 5.1. Cardiovascular-Specific Use of Statins

Currently, the front-line pre-emptive method to address atherosclerosis is the use of statins regardless of LDL levels [121,122,123]. The beneficial effects of statins include not only lipid-lowering, but also immunomodulation and quelling of inflammation [124]. Statins subdue vascular inflammation by reducing expression of IL-6, high-sensitivity C-reactive protein, and homocysteine [125,126]. Their cholesterol-independent pleiotropic effects extend to improving endothelial function and lowering oxidative stress [127]. They inhibit vascular smooth muscle proliferation and platelet aggregation [128]. They modulate the functions of adhesion molecules and reduce monocytes adhesion to endothelial cells. Statins have not been found to change the course of RA itself and thus are given specifically for cardiovascular risk reduction [129].

### 5.2. Drugs That Treat RA and Their Impact on ASCVD

Therapies directed against RA that also mitigate some of the associated CVD risk would be particularly valuable and there are several to be discussed here and summarized in Table 1. Disease-modifying anti-rheumatic drugs (DMARDs), which decrease the inflammation common to RA and ASCVD have a variable track record in atheroprotection. Studies such as TARGET are intended to delve into whether a reduction in inflammation via DMARDs does, in fact, reduce the risk of CVD [120].

#### 5.2.1. Methotrexate

One such DMARD investigated by the TARGET study is MTX, a folic acid analog that inhibits dihydrofolate reductase and reduces the levels of tetrahydrofolate in cells. MTX also inhibits purines and pyrimidines necessary for DNA and RNA synthesis [130]. Another mechanism by which it reduces inflammation is by lowering levels of NF-κB activity [131]. MTX is widely used in the treatment of various inflammatory and autoimmune disorders and it is the initial therapy of choice for RA [132,133]. MTX possesses cardioprotective properties that may reduce CVD morbidity and mortality in patients with RA [134,135,136,137]. 

Low dose MTX, the gold standard RA treatment, broadly inhibits several immune pathways involved in purine and pyrimidine synthesis, transmethylation reactions, translocation of NF-κB to the nucleus, signaling via the Janus kinase (JAK)–signal transducer and activator of transcription (STAT) pathway and nitric oxide production [138]. The benefits of MTX in reducing joint symptoms in RA can be attributed to these anti-inflammatory mechanisms described above. The protective effects of MTX against CVD can occur indirectly via anti-inflammatory actions and also directly via antiatherogenic effects, such as enhancing cholesterol transport out of monocytes and macrophages and preventing the differentiation and activation of foams cells [139,140,141]. Low-dose MTX also promotes release of the anti-inflammatory endogenous nucleoside adenosine, which acts on its receptors to increase production of ABCA1 and ABCG1, proteins involved in cholesterol efflux from macrophages [142]. MTX also upregulates free radical scavenging and improves endothelial function by reducing the expression of adhesion molecules, consequently inhibiting the recruitment of proinflammatory T cells and monocytes [143]. Of note, by depleting tetrahydrofolate, which is needed for conversion of homocysteine to methionine, MTX may have negative effects on cardiovascular health by raising plasma homocysteine levels, thus contributing to oxidative stress [144]. However, folate supplementation can mitigate this rise in homocysteine, promoting an overall anti-atherogenic effect [143].

In a multicenter prospective cohort study of 2044 US veterans with RA, Johnson et al. [145] found that the use of MTX was associated with a significant reduction in CVD related adverse events. In this study, CVD risk reduction by MTX was independent of age, BMI, traditional CVD risk factors, and RA disease activity. The cardioprotective properties of MTX were not solely due to amelioration of RA disease activity, suggesting that MTX may have direct cardiovascular advantages for patients with RA. 

#### 5.2.2. Sulfasalazine

Sulfasalazine, an older anti-rheumatic drug also being investigated in the TARGET study, is a prodrug synthesized from the fusion of the antibiotic sulfapyridine and an NSAID, 5-aminosalicylic acid via an azo bond. This oral sulfonamide drug possesses antithrombotic properties. It inhibits platelet thromboxane synthetase and therefore thromboxane production [146]. Sulfasalazine can prevent arachidonic acid-mediated platelet aggregation, an inhibition equivalent to that produced by aspirin given for protection against CVD [147]. Sulfasalazine decreases proliferation of lymphocytes, and adhesion of monocytes and leukocytes [148]. Sulfasalazine reduces production of inflammatory cytokines, likely through inhibition of NF-κB. As noted previously, NF-κB is a major regulator of genes that promote inflammation and adhesion in cell types involved in atherogenesis [83,84]. Sulfasalazine acts on the NF-κB pathway by preventing the phosphorylation and successive degradation of IκB, the inhibitory subunit of NF-κB [149,150]. Inhibition of NF-κB is considered a viable approach to protection from atherosclerosis development [151].

Sulfasalazine positively impacts the lipid profile, increasing HDL-C and reducing the atherogenic ratio [152,153,154]. Observational data from a QUEST-RA longitudinal cohort study also indicated that longer exposure to sulfasalazine correlated with a reduced risk of CVD events and myocardial infarction [155]. 

#### 5.2.3. TNF-α Inhibitors 

Based upon the pro-atherogenic actions of TNF-α, the reduction in systemic inflammation with the use of biologics such as TNF inhibitors (TNFi) could be expected to contribute to improvements in endothelial function and confer atheroprotection [156,157,158,159]. Commonly used TNF-α inhibitors include etanercept, infliximab and adalimumab [160]. Etanercept is a dimeric fusion protein consisting of the extracellular portion of the human TNF receptor linked to the Fc portion of human immunoglobulin 1 (IgG1). It targets free TNF-α and TNF-β. Infliximab is a chimeric monoclonal antibody with a murine variable region that specifically binds to human TNF-α. Adalimumab is a fully human monoclonal anti-TNF-α antibody. All exert their effect by neutralizing circulating TNF-α.

A 5-year study that compared RA patients who were biologic-naïve receiving only synthetic DMARDs and those taking a TNFi (either etanercept, infliximab or adalimumab) found that the risk of myocardial infarction decreased by 39% in the latter group [161]. Vegh et al. showed that one year of TNFi treatment significantly improved pulse–wave velocity, a marker of arterial stiffness and resulted in no significant increase in common carotid intima-media thickness in a mixed cohort of RA and ankylosing spondylitis patients [162]. Serum levels of biomarkers for atherosclerosis may be reduced by TNFi treatment in RA patients. Pusztai et al. followed 53 RA patients on TNFi for one year and found that this treatment significantly decreased the level of circulating complexes of oxLDL bound to β2-glycoprotein [163]. These complexes can be taken up into macrophages to form foam cells and are associated with the development of acute coronary syndromes [164].

Additionally, higher levels of TNF-α may amplify standard cardiac risk factors such as diabetes and dyslipidemia in patients with RA [165,166]. Insulin resistance has been associated with elevated levels of TNF-α in RA patients [167]. TNF-α promotes insulin resistance by directly suppressing activities of insulin-induced insulin receptor substrate-1 and peroxisome proliferator-activated receptor gamma (PPAR-γ) [165,166]. TNFi increases insulin sensitivity in RA patients compared to patients not receiving biologics [168].

The effects of TNFi on lipid profile are unclear, with some studies showing an increase in LDL with treatment [169]. However, other studies show no effect, and a recent study from Korea found no detrimental impact over a four year period [170,171,172].

#### 5.2.4. IL-6 Inhibitors

The introduction of biologic drugs has considerably improved the outcome for patients with RA, but at the same time certain drugs, specifically the IL-6 inhibitors (i.e., tocilizumab), have been a source of controversy in terms of their effects on cardiovascular health, especially with regard to their effects on the lipid profile [173]. A representative prospective study revealed that in patients undergoing tocilizumab therapy, serum concentrations of total cholesterol, LDL, HDL and triglycerides increased during the first 24 weeks of treatment [174]. The results of several randomized clinical trials also showed increased LDL levels in tocilizumab-treated RA patients, of a magnitude greater than the increase seen in RA patients treated with conventional DMARDs [175,176]. Nevertheless, recent studies have shown encouraging data regarding major adverse cardiovascular events with tocilizumab compared to other biologic DMARDs. Two studies conducted underscored a significant decrease in cardiovascular events with this drug [177,178]. Zhang et al., revealed that tocilizumab reduced the risk of myocardial infarction significantly more than abatacept, another biologic DMARD [177]. Kim et al. indicated better cardiovascular outcomes with tocilizumab when compared to TNFi [178]. Furthermore, tocilizumab decreases inflammatory proteins such as serum amyloid A and may restore the anti-atherogenic function of HDL [179]. Effects on HDL are observed directly in recent studies that found tocilizumab to increase HDL cholesterol efflux capacity in RA [180,181]. Cholesterol efflux capacity is considered a strong negative ASCVD risk determinant [182]. The atherogenic lipoprotein Lp(a) was also reduced by tocilizumab [183,184].

#### 5.2.5. JAK Kinase Inhibitors

JAK inhibitors, small molecules that target the JAK-STAT signaling pathway, are targeted synthetic DMARDs for the treatment of RA and other immune-mediated inflammatory diseases [185,186]. Unlike the protein-based biologic DMARDs, the JAK inhibitors do not cause formation of anti-drug antibodies and can be taken orally [187]. Unfortunately, the JAK inhibitors are associated with risk of venous thromboembolism and pulmonary embolism [188]. The effect of these drugs on cardiovascular risk has not yet been determined as data is inconclusive. Charles-Schoeman et al. pooled data from multiple studies on the first generation JAK inhibitor tofacitinib showed low incidence of cardiac adverse events, comparable to that found with placebo [189]. A further study by Charles-Shoeman with post hoc analysis, after 24 weeks of tofacitinib showed increased HDL levels which was associated with lower cardiovascular event risk [190]. A long-term study over 9.5 years found incidence rates for major adverse cardiovascular event with tofacitinib of 0.4 with events per 100 patient-years, which is comparable to those seen with biologic agents [191]. Upadacitinib, a newer and more selective JAK1 inhibitor, has shown comparable effects on cardiovascular risk to placebo and other JAK inhibitors. It raised both HDL and LDL while leaving the HDL-to-LDL ratio constant [192]. The cardiac event risk from these drugs appears low, but more studies are in progress [193,194]. 

#### 5.2.6. HCQ: A Potential Double Agent

Despite its original intended purpose as an anti-parasitic agent, the therapeutic impact of HCQ extends beyond malaria to multiple autoimmune diseases in which it acts as an immunomodulator and its potential benefits continue to emerge [118,195,196,197]. HCQ can improve the survival rates of patients with various autoimmune diseases including RA and systemic lupus erythematosus (SLE) via its immunosuppressive and anti-inflammatory activity [198,199,200].

HCQ is a weak basic 4-aminoquinoline compound that differs from chloroquine by the placement of a single hydroxyl group [197,198]. This subtle alteration decreases toxicity while conserving efficacy, rendering it safer and therefore preferable to chloroquine [196]. The drug is administered orally, as a sulfate tablet, and appears to be well-tolerated, with efficient absorption, a large volume of distribution and bioavailability, as well as a prolonged half-life of about 40–50 days [201]. Approximately 50% of HCQ remains plasma bound and is metabolized into three metabolites, desethyl-chloroquine, desethyl-hydroxychloroquine, and bis-desethyl-hydroxychloroquine, in the liver.

Although the exact mechanisms of action of this compound remain uncertain, it is postulated that immunosuppression results from its ability to block the stimulation of toll-like receptors, suppress T-cell proliferation, inhibit autophagy and reduce macrophage-mediated cytokine production and calcium signaling in B and T cells [202,203]. Its atheroprotective attributes may be due to the reduction in circulating cytokines such as Il-1, IL-6 and TNF-α [204].

In addition to its role as an anti-rheumatic drug, HCQ has anti-diabetic and cardioprotective capabilities. It improves glycemic control, positively impacts insulin sensitivity and metabolism, favorably influences the lipid profile and has anti-thrombotic and anticoagulant properties [196,198,205,206,207,208]. In RA patients, HCQ improves microvascular endothelial function in RA [209].

Additionally, HCQ is a favorable candidate for not only RA treatment but also the management of CVD in these patients as there are few contraindications and adverse effects. The most common side effects predominantly relate to the gastrointestinal issues, such as nausea, vomiting and diarrhea [210]. Although rare, the possibility of developing retinal, neuromuscular, cardiac, and hematological impairments, including retinopathy, does exist [211]. Despite its limited efficacy in the treatment of RA when used alone, HCQ’s utilization in combination therapies may have a significant impact on the cardiovascular risks associated with this rheumatic disease, thereby reducing the burden for both the individual and society [212].

## 6. Conclusions

One of the most life-threatening complications associated with RA is the development of CVD. RA patients have a 30–50% increased risk of cardiac events compared to the general population [15,16]. CVD may go undiagnosed and untreated for prolonged periods in RA patients as it is often subclinical and asymptomatic. Factors responsible for elevated CVD rates in RA include traditional CVD risk factors and an environment induced by RA of persistent inflammation and oxidative stress with constant exposure to high levels of inflammatory cytokines. The excess mortality resulting from ASCVD makes it imperative that consideration be given to the cardiovascular system when developing an RA treatment plan. Despite the clear need to include heart health in clinical management of RA, there has been little progress in this respect and the ongoing primary goal of RA therapies is to achieve disease remission (focused on joints) or at least low disease activity. Cardiovascular-specific management of ASCVD in RA is often limited to use of statins to improve lipid profile and anti-hypertensive medications in patients with elevated blood pressure. Several anti-rheumatic drugs possess established or likely cardiovascular benefits. These include methotrexate, sulfasalazine, TNF-α inhibitors, Il-6 inhibitors and hydroxychloroquine. The ongoing TARGET study holds promise because it is designed to explore which combination of DMARDs will best reduce the risk of CVD in RA. More such prospective trials are needed, and it is hoped that new therapies will bring life-prolonging outcomes for the RA population.

## Figures and Tables

**Table 1:** Therapies Effective Against RA and ASCVD.

DMARDs		
Name of Drug	Mechanism of Action	
	Anti-rheumatic Properties	Atheroprotective Properties
Methotrexate	Inhibits dihydrofolate reductase and several immune pathways involved in purine and pyrimidine synthesis.	Enhances macrophage cholesterol efflux and prevents foams cell differentiation and activation. Upregulates free radical scavenging; improves endothelial function.
Sulfasalazine	Reduces production of inflammatory cytokines, likely through inhibition of NF-κB activation.	Prevents arachidonic acid-mediated platelet aggregation, decreases adhesion of monocytes and leukocytes, and increases HDL-C.
Hydroxychloroquine	Interferes with toll-like receptor signaling, reduces calcium signaling in B and T cells and matrix metalloprotease activity	Positively impacts insulin sensitization, promotes anti-atherogenic lipid profile. Anti-thrombotic and anticoagulant properties.
Tumor Necrosis Factor (TNF)-α Inhibitors		
Etanercept Infliximab Adalimumab	Biologics that inactivate TNF-α. Etanercept is a fusion protein of human immunoglobulin 1 Fc domain and TNF-α receptor. Infliximab is a mouse-human chimeric anti-human TNF-α antibody Adalimumab is a human anti-human TNF-α antibody	TNF-α promotes numerous inflammatory responses associated with atherosclerosis, including induction of vascular adhesion and monocyte/macrophage proliferation. TNF-α impacts lipid metabolism by stimulating liver triglyceride production.
IL-6 Inhibitors		
Tocilizumab	Inhibits IL-6 which contributes to inflammation and antibody production through its action on T cells, B cells, monocytes and neutrophils	Decreases inflammatory proteins such as serum amyloid A, and restores the anti-atherogenic function of HDL by increasing HDL cholesterol efflux capacity.
JAK Kinase Inhibitors		
Tofacitinib	Small molecules that target the JAK-STAT signaling pathway. Reduce expression of cytokine related genes.	Risk of adverse cardiovascular events still being evaluated. Many studies show no difference compared to placebo or biologic
Upadacitinib	More JAK1 selective	

## Data Availability

Data sharing not applicable.
